# Late Cystoid Macular Edema Following Treated Bilateral Endogenous Endophthalmitis Due to Klebsiella pneumoniae: A Case Report

**DOI:** 10.7759/cureus.111047

**Published:** 2026-06-17

**Authors:** Youssef Ennassimi, Sarah Belghmaidi, Younes Tlemcani, Abdeljalil Moutaouakil

**Affiliations:** 1 Department of Ophthalmology, Centre Hospitalier Universitaire Mohammed VI, Marrakech, MAR; 2 Department of Ophthalmology, Faculty of Medicine and Pharmacy, Centre Hospitalier Universitaire Mohammed VI, Université Cadi Ayyad, Marrakech, MAR

**Keywords:** cystoid macular edema, endogenous endophthalmitis, hepatic abscess, klebsiella pneumoniae, optical coherence tomography, sub-tenon triamcinolone acetonide, uveitic macular edema

## Abstract

Endogenous endophthalmitis (EE) is a rare but potentially devastating intraocular infection resulting from hematogenous seeding of an intraocular pathogen from a distant infectious focus. *Klebsiella pneumoniae* is an increasingly recognized causative agent, classically associated with hepatic abscesses, particularly in Asian populations, but with a growing incidence worldwide. Despite adequate treatment, late inflammatory complications may occur; cystoid macular edema (CME) as a delayed sequela of treated EE has been rarely described in the literature. We report a 64-year-old woman with no prior ophthalmic history and a documented penicillin allergy who presented with bilateral EE secondary to *K. pneumoniae* hepatic abscesses. At presentation, visual acuity (VA) was counting fingers at one meter in the right eye (RE) and no light perception in the left eye (LE). Treatment included intravenous (IV) imipenem, ciprofloxacin, and metronidazole, combined with bilateral intravitreal injections of vancomycin and ceftazidime. Microbiological analysis of hepatic drainage fluid confirmed *K. pneumoniae* on day 5. At discharge, RE VA recovered to 8/10, while the LE required evisceration due to irreversible functional loss. Two months later, the patient presented with a sudden drop in RE VA to 1/10. Macular optical coherence tomography (OCT) revealed confluent cystic intraretinal spaces with a central macular thickness (CMT) of 620 μm and a macular volume of 11.35 mm³, consistent with inflammatory CME. After confirming infection control, two posterior sub-Tenon injections of triamcinolone acetonide (40 mg/1 mL each), spaced one month apart, resulted in significant edema regression (CMT: 408 μm, average thickness: 341 μm) with VA improvement to 6/10. This case illustrates the occurrence of delayed CME as a late complication of treated EE, likely sustained by persistent subclinical inflammation mediated by pro-inflammatory cytokines (vascular endothelial growth factor (VEGF), interleukin-6 (IL-6), and tumor necrosis factor-alpha (TNF-α)), disrupting the blood-retinal barrier (BRB). Sub-Tenon triamcinolone acetonide proved effective and well-tolerated. Prolonged ophthalmologic follow-up with systematic macular OCT is warranted after treated EE to enable early detection and management of late inflammatory complications such as CME.

## Introduction

Endogenous endophthalmitis (EE) is a severe intraocular infection that arises from the hematogenous dissemination of microorganisms from a systemic infectious source. It accounts for 2%-8% of all endophthalmitis cases and carries a poor visual prognosis, particularly in its bilateral form [1,2]. Among causative agents, *Klebsiella pneumoniae* occupies a prominent position owing to its predilection for hepatic abscesses. Although historically reported predominantly in Southeast Asian populations, this organism is increasingly implicated in EE cases across non-endemic regions [2,3].

The acute management of EE typically relies on systemic and intravitreal antimicrobial therapy, with vitrectomy reserved for severe or refractory cases [4,5]. However, even after successful eradication of the infectious focus, late-onset inflammatory complications may arise. Among these, cystoid macular edema (CME) represents a particularly sight-threatening complication that has been described in the context of uveitis but remains poorly documented as a sequela of treated bacterial EE [4,6].

We report a case of bilateral EE secondary to *K. pneumoniae* hepatic abscesses in which late CME occurred two months after clinical resolution, successfully managed with posterior sub-Tenon triamcinolone acetonide injections. This observation underscores the importance of prolonged ophthalmic surveillance following treated EE.

## Case presentation

A 64-year-old woman with no prior ophthalmic history and a documented allergy to penicillin presented to our emergency department on August 6, 2024, with a painful, red left eye (LE) associated with high-grade fever (40°C). Two days after admission, involvement of the right eye (RE) was noted. On examination, VA was limited to counting fingers at one meter in the RE and absent (no light perception) in the LE. Slit-lamp examination revealed bilateral conjunctival hyperemia, a Tyndall effect graded 2+ in the RE and 4+ in the LE, chemosis with purulent discharge in the LE, and dense bilateral hyalitis precluding fundoscopic assessment in both eyes.

**Figure 1:**
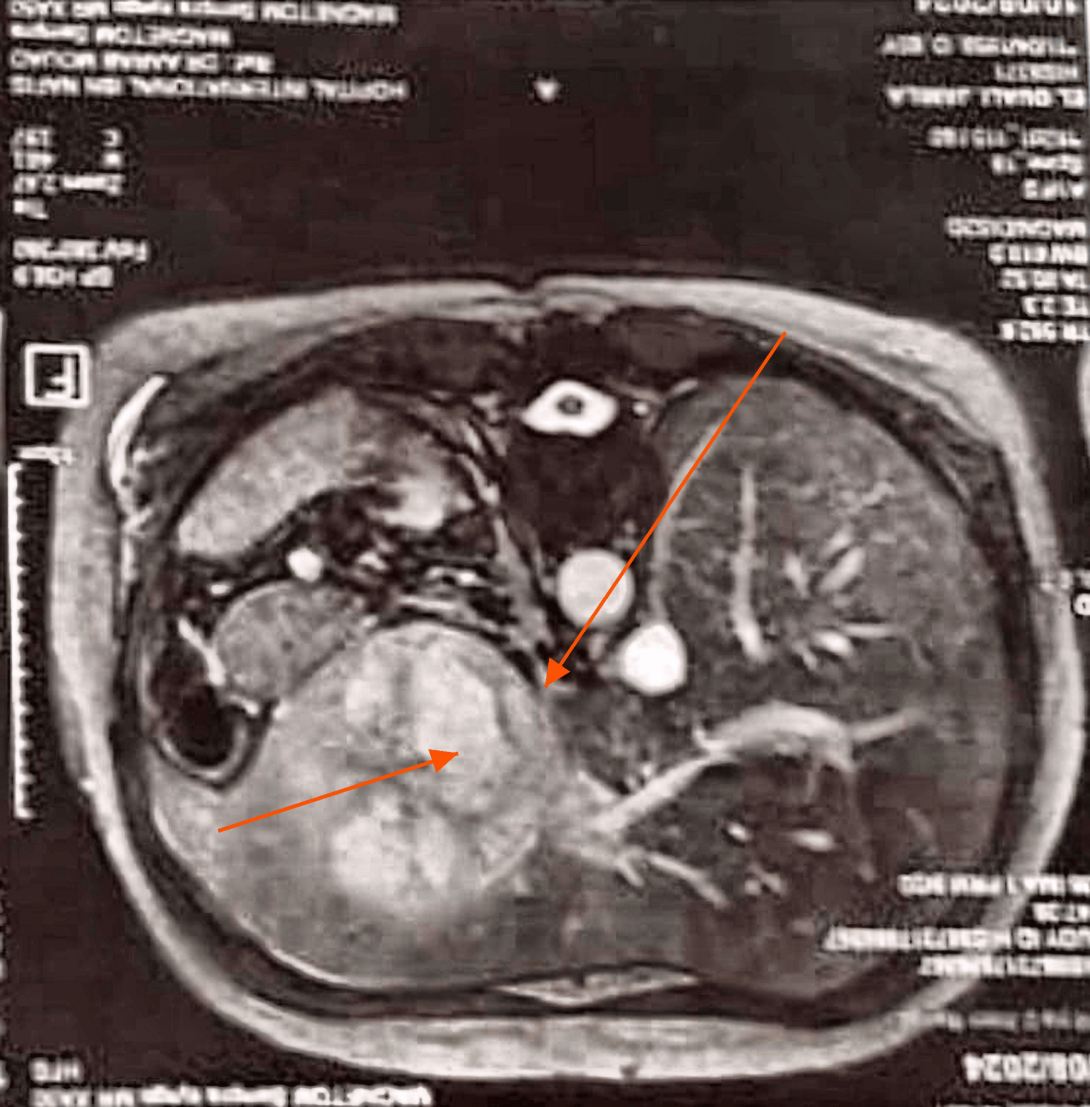
Representative axial T2-weighted MRI image demonstrating a multiloculated hepatic abscess with heterogeneous content (arrow), representing the systemic infectious focus responsible for hematogenous endogenous dissemination. Microbiological analysis of the drainage fluid isolated *Klebsiella pneumoniae*. MRI: magnetic resonance imaging

B-scan ultrasonography confirmed bilateral vitreous involvement and identified a subretinal collection in the LE. Hepatic magnetic resonance imaging (MRI) disclosed multiloculated hepatic abscesses (Figure 1). Blood cultures were negative; however, microbiological analysis of hepatic drainage fluid, collected on day 5, isolated *Klebsiella pneumoniae*, confirming the endogenous origin of the endophthalmitis. No intravitreal sampling was performed, given the septic context, the availability of systemic microbiological confirmation, and the favorable initial response of the RE to treatment.

Treatment consisted of intravenous (IV) imipenem (500 mg every 6 hours), ciprofloxacin (400 mg every 12 hours), and metronidazole (500 mg every 8 hours), along with three bilateral intravitreal injections of vancomycin (1 mg/0.1 mL) and ceftazidime (2 mg/0.1 mL). Intravitreal voriconazole (100 μg/0.1 mL) was added empirically to cover possible fungal etiology and was discontinued upon microbiological confirmation of a bacterial pathogen. Surgical drainage of the hepatic abscesses was performed on day 5. Vitrectomy was not undertaken, given the patient’s systemic condition and the satisfactory early response of the RE.

At discharge, RE VA had recovered to 8/10 (0.1 logMAR). The LE showed no functional recovery and required evisceration.

**Figure 2:**
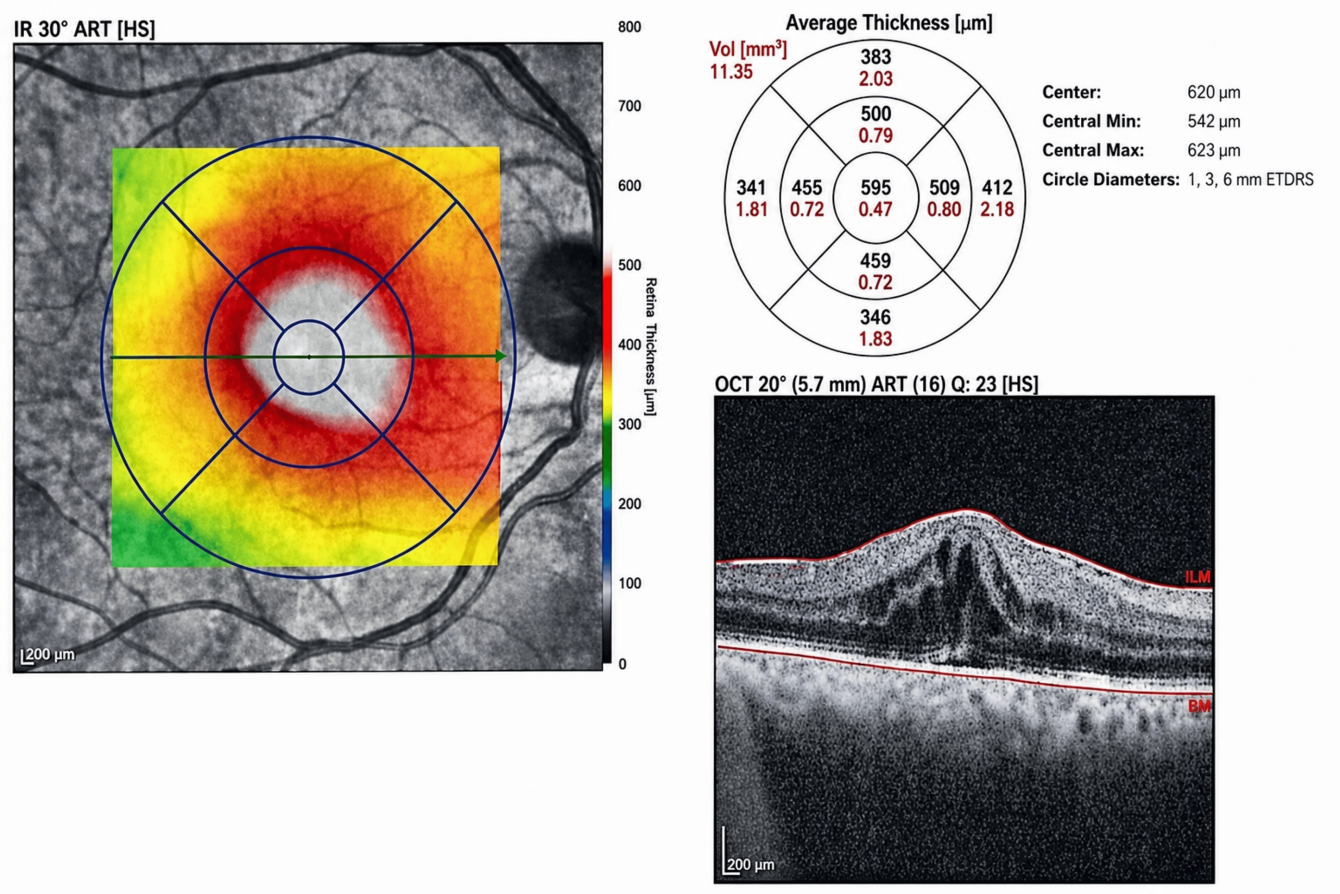
Baseline macular optical coherence tomography (OCT) of the right eye, two months after the acute episode and before treatment. Early Treatment Diabetic Retinopathy Study (ETDRS) thickness map demonstrating a central macular thickness of 620 µm (macular volume: 11.35 mm³) with intense foveal hyperthickening, consistent with severe cystoid macular edema.

Two months after discharge, the patient returned with a sudden decrease in RE VA to 1/10. The anterior segment was quiet, with iridocorneal posterior synechiae and early posterior subcapsular cataract. Macular optical coherence tomography (OCT) demonstrated confluent cystic intraretinal spaces, with a central macular thickness (CMT) of 620 μm and a macular volume of 11.35 mm³ (Figure 2), consistent with inflammatory CME. Systemic infection control was verified prior to any anti-inflammatory intervention (apyrexia, normalized C-reactive protein, quiet anterior segment, and absence of vitritis).

**Figure 3:**
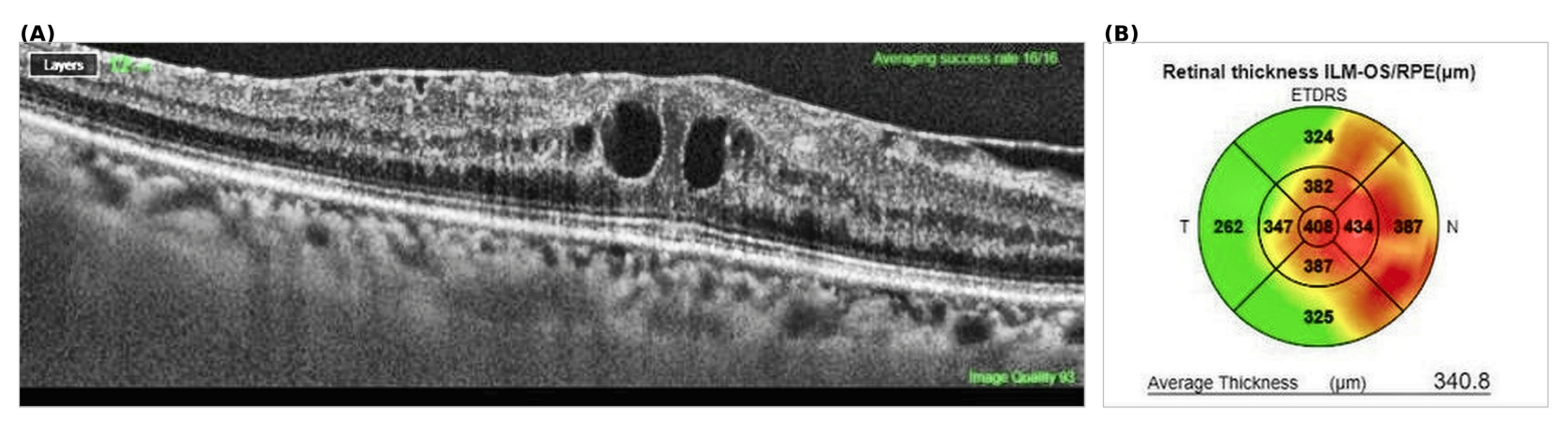
Follow-up macular optical coherence tomography (OCT) of the right eye after two posterior sub-Tenon triamcinolone acetonide injections (40 mg/1 mL each), spaced one month apart. (A) Representative B-scan demonstrating significant reduction of cystic intraretinal spaces with partial restoration of foveal architecture; the arrow indicates the area of residual macular edema. (B) Internal Limiting Membrane to Outer Segment/Retinal Pigment Epithelium (ILM-OS/RPE) ETDRS thickness map showing significant edema regression: central macular thickness 408 µm, average macular thickness 340.8 µm. Visual acuity: 6/10 (0.2 logMAR).

Two posterior sub-Tenon injections of triamcinolone acetonide (40 mg/1 mL each), spaced one month apart, were administered with intraocular pressure monitoring [7]. Follow-up macular OCT demonstrated significant edema regression, with a CMT of 408 μm and an average macular thickness of 341 μm, associated with improvement in RE VA to 6/10 (0.2 logMAR) (Figure 3). Angio-OCT performed at follow-up revealed residual vascular disturbances in the superficial and deep capillary plexuses, consistent with resolving inflammation.

## Discussion

This case illustrates a rare but clinically significant complication: the occurrence of CME two months after apparent resolution of bilateral *K. pneumoniae* EE. To our knowledge, delayed CME as a late sequela of treated bacterial EE has been only rarely reported, making this observation a noteworthy contribution to the existing literature [4,5].

Epidemiology and clinical features of *K. pneumoniae* EE

EE accounts for 2%-8% of all forms of endophthalmitis and results from hematogenous seeding of the eye from a systemic infectious focus [1,2]. *Klebsiella pneumoniae* is the leading causative agent in Southeast Asia, where it has been strongly associated with primary hepatic abscesses in otherwise immunocompetent individuals [3]. Its incidence is rising in non-Asian populations, and our case confirms that clinicians outside endemic regions should maintain awareness of this entity [2,3]. Bilateral involvement, as observed here, portends a particularly unfavorable visual prognosis; the LE in our patient ultimately required evisceration [1,2].

Pathophysiology of late CME

Post-inflammatory CME results from disruption of the blood-retinal barrier (BRB) driven by pro-inflammatory mediators, particularly vascular endothelial growth factor (VEGF), interleukin-6 (IL-6), and tumor necrosis factor-alpha (TNF-α), whose pathogenic roles are well established in uveitis-associated macular edema [4,8]. In our patient, the onset of CME two months after clinically resolved EE suggests the persistence of subclinical intraocular inflammation despite the absence of overt infectious or inflammatory signs. This “smoldering” inflammatory state may result from residual intraocular cytokine activity, impaired BRB reconstitution following acute insult, or inflammatory response to residual antigenic material. The absence of active vitritis at presentation of CME, combined with a normalized inflammatory workup, argued against active infection and supported the diagnosis of post-inflammatory CME.

Treatment and outcome

Sub-Tenon triamcinolone acetonide has demonstrated efficacy in uveitic macular edema in several studies, offering sustained local corticosteroid delivery with a lower risk of systemic side effects compared with systemic steroids, and a reduced risk of significant intraocular pressure elevation and endophthalmitis compared with intravitreal injections [7]. In our patient, two injections administered one month apart led to a meaningful reduction in CMT (from 620 μm to 408 μm) and a clinically significant improvement in VA (from 1/10 to 6/10). The persistence of residual vascular disturbances on angio-OCT at follow-up suggests ongoing, albeit regressing, microvascular inflammation and may explain the incomplete visual recovery. Additional contributing factors include posterior synechiae and early cataract, both of which may further impair visual outcomes. The incomplete visual recovery to 6/10 may also reflect photoreceptor damage sustained during the acute infectious episode, a common sequela in severe bacterial EE [5,9].

Clinical implications

This case highlights two key clinical messages. First, late CME should be considered in the differential diagnosis of visual decline following treated EE, even in the absence of clinical signs of active infection or inflammation. Second, posterior sub-Tenon triamcinolone acetonide represents an effective and safe therapeutic option in this setting, provided adequate infection control has been confirmed.

Limitations

The principal limitations of this report include the absence of intravitreal sampling (which would have provided direct microbiological confirmation of intraocular infection), the use of two different OCT platforms at baseline and follow-up (precluding fully quantitative comparison of thickness measurements, with analysis therefore remaining primarily qualitative), and the single-case nature of the observation, which limits generalizability.

## Conclusions

This case demonstrates that CME may occur as a delayed, sight-threatening complication of treated EE due to *K. pneumoniae*, even in the absence of active infection or overt inflammation. Prolonged ophthalmic surveillance with systematic macular OCT following treated EE is strongly recommended to enable early identification and treatment of such complications. Posterior sub-Tenon triamcinolone acetonide represents an effective therapeutic approach in this context.
